# Physician assessments of medication adherence and decisions to intensify medications for patients with uncontrolled blood pressure: still no better than a coin toss

**DOI:** 10.1186/1472-6963-12-270

**Published:** 2012-08-21

**Authors:** Jennifer Meddings, Eve A Kerr, Michele Heisler, Timothy P Hofer

**Affiliations:** 1 Department of Internal Medicine, University of Michigan, Ann Arbor, USA; 2 Center for Clinical Management Research, VA Ann Arbor Healthcare System, Ann Arbor, USA

**Keywords:** Adherence, Hypertension, Diabetes, Veterans, Quality improvement

## Abstract

**Background:**

Many patients have uncontrolled blood pressure (BP) because they are not taking medications as prescribed. Providers may have difficulty accurately assessing adherence. Providers need to assess medication adherence to decide whether to address uncontrolled BP by improving adherence to the current prescribed regimen or by intensifying the BP treatment regimen by increasing doses or adding more medications.

**Methods:**

We examined how provider assessments of adherence with antihypertensive medications compared with refill records, and how providers’ assessments were associated with decisions to intensify medications for uncontrolled BP. We studied a cross-sectional cohort of 1169 veterans with diabetes presenting with BP ≥140/90 to 92 primary care providers at 9 Veterans Affairs (VA) facilities from February 2005 to March 2006. Using VA pharmacy records, we utilized a continuous multiple-interval measure of medication gaps (CMG) to assess the proportion of time in prior year that patient did not possess the prescribed medications; CMG ≥20% is considered clinically significant non-adherence. Providers answered post-visit Likert-scale questions regarding their assessment of patient adherence to BP medications. The BP regimen was considered intensified if medication was added or increased without stopping or decreasing another medication.

**Results:**

1064 patients were receiving antihypertensive medication regularly from the VA; the mean CMG was 11.3%. Adherence assessments by providers correlated poorly with refill history. 211 (20%) patients did not have BP medication available for ≥ 20% of days; providers characterized 79 (37%) of these 211 patients as having significant non-adherence, and intensified medications for 97 (46%). Providers intensified BP medications for 451 (42%) patients, similarly whether assessed by provider as having significant non-adherence (44%) or not (43%).

**Conclusions:**

Providers recognized non-adherence for less than half of patients whose pharmacy records indicated significant refill gaps, and often intensified BP medications even when suspected serious non-adherence. Making an objective measure of adherence such as the CMG available during visits may help providers recognize non-adherence to inform prescribing decisions.

## Background

Non-adherence with blood pressure (BP) medications is commonly cited as a cause of uncontrolled hypertension, and BP medication non-adherence is associated with worse outcomes [1,2]. Physicians frequently need to decide if the patient’s BP is too high because the patient isn’t taking medications as prescribed, or if the patient’s current BP prescription needs intensification. Unfortunately, healthcare providers can have difficulty making this decision because BP medication adherence problems can be difficult for providers to detect [3-6]. Adherence problems lead to suboptimal control of hypertension in two ways: the patient does not take medications as prescribed and the possibility of non-adherence increases the uncertainty faced by physicians in deciding medication changes [7,8]. This common clinical quandary inspired the following research questions:

1. How often do providers correctly identify patients with poor adherence to chronic BP medications?

2. When BP is uncontrolled, how often do providers intensify the medication regimen for patients that providers believe have significant non-adherence with BP medications?

To address these questions, we used data from the ABATe (Addressing Barriers to Treatment of Hypertension) study [9], designed to examine treatment change decisions for diabetic primary care patients with elevated triage BP (≥  140/90) at primary care visits. We hypothesized that many patients who presented with uncontrolled BP would have pharmacy refill patterns that supported non-adherence by late refill requests. Because non-adherence can be difficult for providers to recognize [3-5], we also hypothesized that many primary care providers would not recognize non-adherence in their patients. Yet, we expected that when primary care providers *did* recognize significant medication non-adherence, they would be *less* likely to respond to elevated BP by intensifying the medication regimen by either increasing the dose(s) or prescribing extra medications.

## Methods

### Design and participants

**Figure 1:**
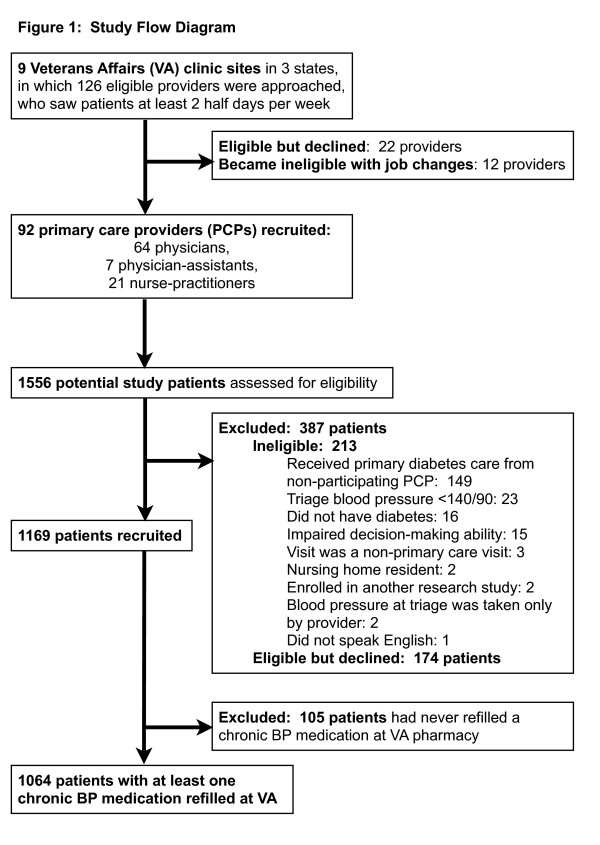
Study flow diagram.

We performed an analysis of medication adherence using information collected in a larger cross-sectional cohort study [9] of patients scheduled for primary care visits with 92 primary care providers at 9 Department of Veterans Affairs (VA) facilities of varied sizes in 3 mid-western states. The institutional review boards of all participating VA facilities approved the study protocol; the VA institutional review board policies are based on The Common Rule (45 CFR 46, Subpart A) governing research, rooted in several US and international research ethics guidelines, including the Helsinki Declaration. All recruited patients had diabetes, their lowest BP in triage was ≥140/90, and the patients had identified the VA as their primary source of care for diabetes. Of note, by clinic triage policies, a second blood pressure measurement should be obtained if the first blood pressure was elevated; patients were eligible if their lowest triage systolic blood pressure was 140 mmHg or greater or if their lowest diastolic blood pressure was 90 mmHg or greater. Patients with impaired decision-making ability, terminal disease, residents of nursing homes, and those who did not speak English were excluded. As detailed in Figure 1, of the 1556 patients approached by study staff, 213 were ineligible; 1169 patients and their providers signed written consent. The analyses in this paper focus on the 1064 patients who had at least one chronic BP medication refilled at the VA.

### Data sources

Our analyses utilized 4 data sources. First, VA automated data sources were used to obtain BP values at the time of study enrollment. Second, VA pharmacy records for 1 year prior and 90 days after enrollment provided the total number of medications, and the number and refill history for antihypertensive medication classes. Third, providers completed a brief survey for each patient after the same clinic session in which they saw the patient (completion rate, 99%). Fourth, patients completed a brief questionnaire after the visit including self-report of socio-demographic characteristics including age, race/ethnicity, annual income, and monthly prescription costs.

### Measurements

#### 1. Refill adherence measure

For our refill adherence measure, we used the Continuous Multiple-interval Gap (CMG) measure because it has been used previously to assess VA pharmacy data including antihypertensive medications [10-12], and has been assessed in comparison with electronic pill cap monitoring studies [13] which is as close to a ‘gold standard’ of medication adherence currently possible regarding hypertension treatment. In brief, the CMG measure uses pharmacy data (including fill and refill dates, quantities, daily doses) to determine how many days over the past year the patient did not possess BP medications to take as prescribed, expressed as a percentage of days in past year. For example, a CMG of 20% means the patient refilled the medication in a manner that left 20% of days without an adequate medication supply to take. The CMG can also be interpreted as a proportion of time the patient misses their medications, with a CMG of 20% meaning that on average, the patient missed 20% of the doses, or 1 dose in 5. Prior literature has established that a lack of medication possession of  ≥  20% is clinically significant refill non-adherence [11,14,15]. Of note, for medications prescribed for multiple doses per day, it is possible that a patient could still be taking some but inadequate doses of medication each day. For example, a patient taking a twice daily antihypertensive medication could have a CMG of 50% if they are only refilling it in a manner to take it once daily in a regular fashion, or could have been taking it twice daily until they simply stopped taking and refilling the medication half way through the prescribed refills, taking zero medications daily after no longer refilling. For each patient, a CMG was calculated for each class of antihypertensive medications (including potentially 11 classes, such as ace-inhibitors, thiazide diuretics, and beta-blockers). Next, a composite CMG (as a continuous measure) was calculated for all the antihypertensive medications, excluding loop diuretics because this class could be taken for reasons beyond BP control (such as congestive heart failure). Further details of the algorithm we used to calculate the CMG are provided in the Appendix including adjustment for automatic first fills of a new prescription by VA pharmacies. A dichotomous measure was also created to identify patients with significant non-adherence refill gaps of CMG  ≥  20% (versus <20%).

#### 2. Provider assessment of patient’s medication adherence

The primary care providers completed a post-visit questionnaire. This study focused on their responses to 2 questions requiring them to assess the patient’s adherence to BP medications.

In the first question, providers were asked “how often does your patient adhere to the BP regimen?” with scoring options on a Likert scale from 1 (“none of the time”) to a score of 5 (“all of time”). In the second question, providers were asked “how much does adherence make it difficult to control this patient’s BP?” with scoring options from 1 (“not at all”) to a score of 5 (“a great deal”). To generate a continuous measure of provider assessment of adherence, the responses to these questions were summed for a total score of 2–10, after reverse scoring for similar directionality, and imputation of a few missing responses by recording the same level of score from an answered question to be the response to the unanswered question. If the provider had not answered either question but the patient was taking BP medications, the questions were imputed by scoring the average response (3) for both questions.

Next we generated a dichotomous version of this in which the provider was given credit for recognizing significant non-adherence if a provider reported the patient was taking medications “none of time” (score 1) or close to it (score 2), or if the provider indicated that adherence made the patient’s BP difficult to control by “a great deal” (score 5) or close to it (score 4). Providers chose “don’t know” or provided no assessment of adherence for 22 (8%) patients; no imputation was used to generate this dichotomous variable.

#### 3. Provider decision to intensify BP medications

In the post-visit survey, providers also answered questions regarding if and how the antihypertensive regimen was changed at the visit; medication *intensification* (as a dichotomous measure) was defined as providers adding or increasing a BP medication dose without stopping another medication or decreasing a dose [9]. For example, if the provider indicated on the post-visit survey that she had started a new BP medication but also discontinued a different BP medication, this would not count as intensification. However, intensification would have occurred if the provider started a new BP medication but did not either discontinue or decrease another BP medication.

#### Statistical analyses

The primary analyses involved how providers assessed each patient’s medication adherence in comparison to each patient’s adherence according to refill records. First, using continuous measures of provider assessment and refill adherence, the strength of linear correlation between the 2 measures was assessed. Then, using the dichotomized measures for patients with significant non-adherence, we assessed the concordance (or agreement) between categorization of the pharmacy refill score as non-adherent (CMG  ≥  20%) and being identified by the provider as having significant non-adherence. We used a 2-sample *t*-test (accounting for unequal variances) to assess significant differences between these 2 measures.

Our secondary analyses assessed the strength of association between the provider’s decision to intensify the BP medication regimen (as a dependent, dichotomous outcome) with the provider’s assessment of the patient’s adherence (as an independent measure), explored using both logistic regression (using the continuous measure of provider assessment) and chi square statistics (using the dichotomous measure of provider assessment). Covariates studied included the patient’s systolic and diastolic BPs at triage, number of chronic antihypertensive classes being prescribed at time of enrollment, total number of chronic medication classes that a patient was prescribed in the 90 days prior to enrollment and patient self-reported characteristics of age, ethnicity, education, annual income, monthly prescription costs, and marital status.

All statistical analyses were performed using Stata version 11.2 (Stata, College Station, Texas).

## Results

### Participant characteristics

Figure 1 outlines the study population recruitment of providers and patients. As summarized in the Table 1, the lowest systolic BP of patients at enrollment had a mean of 154 mmHg (S.D. 14) and the lowest diastolic BP of patients at enrollment was a mean of 78 mmHg (S.D. 12). The mean age of the 1064 patients was 65 years (S.D. 11); 97% were men, 71% were white, and 54% were married. Overall, the majority (76%) of patients reported an annual income of < $40,000. Monthly prescription costs were reported in a range of $0 (23%) to > $200 per month (6%), with 39% of patients reporting monthly prescription costs of > $50 per month. Patients were taking a mean of 2.9 classes of BP medications, and an average of 6.3 prescription medications for all conditions at time of study enrollment. Ninety-two primary care providers participated with a mean age of 48, and a mean of 11.4 years in practice (range 1–33) including 64 physicians, 21 nurse practitioners, and 7 physician assistants [9]; providers had a median of 12 patients (range of 1–14) in the analyzed study population.

**Table 1:** Patient characteristics

**N, % of patients**	**1064, 100%**	
**Demographics, by self-report**	**Mean +/− SD**	**Not answered: N (%)**
	N, %*	
**Age (range 33–88 years)**	Mean: 65 +/− 11	Not answered:107 (10%)
<45	16, 2%	
≥ 45 & <65	464, 44%	
≥ 65 & <75	257, 24%	
≥ 75	220, 21%	
**Race**		Not answered: 96 (9%)
White	752 (71%)	
Other	216 (20%)	
**Education**		Not answered: 104 (10%)
<High school	168 (16%)	
High School / GED	350 (33%)	
Some college or Trade School	337 (32%)	
4 year degree	56 (5%)	
Post-college	49 (5%)	
**Annual Income by self-report ($)**		Not answered: 162 (15%)
≤10,000	158 (15%)	
>10,000 & ≤ 20,000	287 (27%)	
>20,000 & ≤ 30,000	251 (24%)	
>30,000 & ≤ 40,000	112 (11%)	
> 40,000	94 (9%)	
**Clinical characteristics, by medical record**		
**Systolic Blood Pressure (BP):**	Mean: 154+/−14	No missing data.
>180	56 (5%)	
160-180	252 (24%)	
150-159	274 (26%)	
140-149	455 (43%)	
<140	27 (3%)	
**Diastolic Blood Pressure:**	Mean: 78 +/− 12	No missing data.
>100	31 (3%)	
90-100	164 (15%)	
80-89	281 (26%)	
<80	588 (55%)	
**Number of BP med classes**		No missing data.
1-2	472 (44%)	
3-4	455 (43%)	
≥ 5	137 (13%)	

* Percentages are rounded, so may not total 100%.

### Refill adherence scores (CMG)

**Figure 2:**
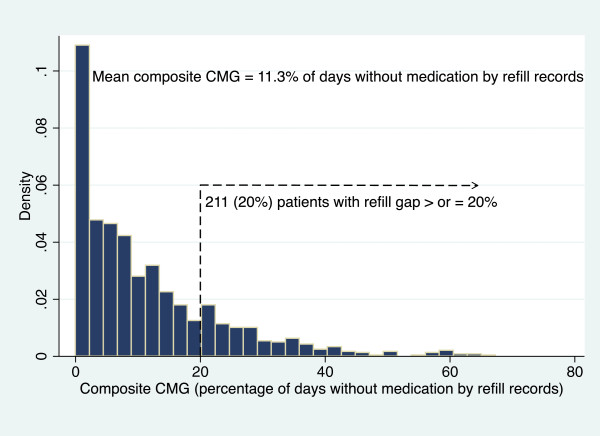
**Provider Assessment of Adherence(Histogram).**

The mean composite CMG for all BP medications for our 1064 patients was 11.3% (S.D. 12%), signifying that, on average, patients did not have adequate medication supply to take as prescribed for 11% of days, or missed 11% of doses. As illustrated in the Figure 2, the distribution of the CMG scores was highly skewed with 80% (N = 853) having a CMG score of < 20%, supporting good refill adherence for most patients in this study population of Veterans with diabetes. Thus, only 211 patients (20%) were categorized as significantly non-adherent by their pharmacy refill scores with CMG ≥ 20%. Only 18 patients (1.7%) had a CMG score indicating anticipated lack of supply for  ≥  50% of days/doses.

### Provider assessment of patient’s medication adherence

**Figure 3:**
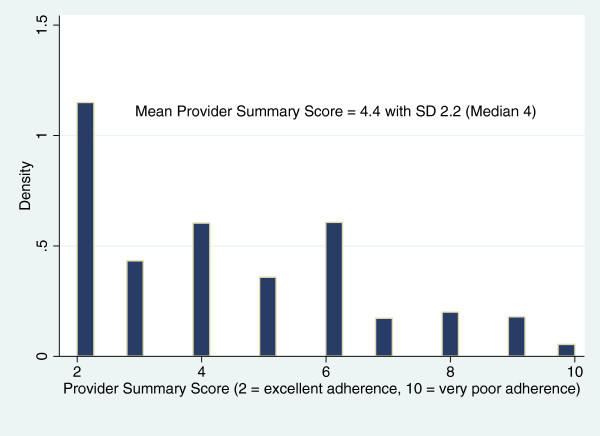
Comparing Identification of Significant Non-adherence by Provider Assessment and Refill History (CMG= Continuous Multiple-interval Gap measure).

Provider responses to each of the 2 questions regarding medication adherence had strong correlation (r = 0.7, p < 0.0001) between questions, before and after imputation of missing responses. Using the continuous summary measure ranging from 2 (suggesting excellent adherence) to 10 (suggesting very poor adherence), provider assessments overall suggested good adherence, skewed toward lower scores (illustrated in Figure 3). Of interest, provider assessments of adherence of non-white patients was somewhat higher (indicating worse adherence) with mean score of 4.92 (95% CI = 4.35-5.48) compared to provider assessments of white patients with a mean score of 4.07 (95% CI = 3.87-4.28) even after adjusting for patient age, refill adherence (CMG), and systolic BP. Providers assessed 258 (24%) patients as having significant non-adherence (using the dichotomous measure) with BP medications by either post-visit question.

### Comparing provider assessment with refill adherence scores (CMG)

Overall, there was weak correlation (r = 0.18, p < 0.001) between the patient’s refill adherence score and the provider’s assessment of patient adherence.

**Figure 4:**
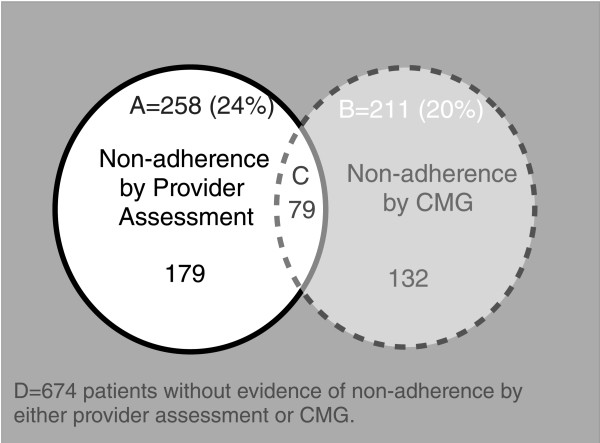
**Comparing Identification of Significant Non-adherence by Provider Assessment and Refill History (CMG= Continuous Multiple-interval Gap measure).Area A** (circle outlined with solid border): 258 (24%) patients identified by provider as having significant non-adherence. **Area B** (circle outlined by dashed border): 211 (20%) patients identified by refill measure (CMG ≥20%) as having significant non-adherence. **Area C** (overlap of areas A and B): 79 (7%) patients identified as having significant non-adherence by both the provider assessment and refill measure (CMG). **Area D** (surrounding gray box): 674 patients without evidence of non-adherence by either provider assessment or refill measure (CMG), including 22 patients for whom the providers did not provide an adherence assessment by either post-visit question.

Using dichotomized measures, a similar proportion of patients was categorized as non-adherent by the gap (CMG ≥ 20%) in pharmacy refills (N = 211, 20%) and by providers (N = 258, 24%); yet, the dichotomized CMG measure and the provider assessment were often identifying significant non-adherence for different patients, as illustrated in Figure 4. Providers appeared to recognize significant non-adherence for only 79 (37%) of the 211 patients who had a CMG ≥ 20%. Among the 18 patients with very large medication refill gaps (CMGs ≥ 50%), providers assessed 8 (44%) as non-adherent. Overall, the mean CMG for patients categorized by providers as having significant non-adherence was 14% (SD 14%), compared with the mean CMG of 10% for patients (SD 11%) for those not identified by providers with significant adherence problems. Quantitative analysis for agreement for the dichotomous categorization of the patient as having poor adherence or not, as indicated by the provider and pharmacy record, were also evaluated using kappa statistics [16]. In general, there was only slight agreement between the categorization by provider and pharmacy record (kappa 0.15).

### Provider decisions to intensify BP medications

Overall, providers intensified BP medications for 451 of the 1064 patients (42%) at the study visit. Using logistic regression with the provider decision to intensify as the dependent variable, the provider’s assessment of adherence (as a continuous, independent variable) was not predictive of the decision to intensify (OR 0.96, p = 0.25). Of the covariates studied (systolic BP, age, race, refill gap), only higher systolic BP (per 10 mm-Hg pressure increase) was significantly associated with the provider’s decision to intensify medications (OR 1.40, p < 0.0001, 95% CI 1.26-1.55).

Of the 258 patients identified by the provider as having significant non-adherence, providers intensified medications for 113 (44%) of these patients; similarly, providers intensified medications for 42% of patients that were not identified by providers as having significant non-adherence. Overall, there was no significant difference in providers’ decisions to intensify medications whether providers had assessed patients as adherent or not.

## Discussion

In this sample, primary care providers’ assessments of adherence did not agree with more objective pharmacy fill assessments about half of the time. In general, providers overestimated adherence with antihypertensive medications, and were still less accurate in identifying cases of significant non-adherence than a coin toss [17]. Unawareness and/or inaccuracy of physician assessment of adherence to antihypertensiveshas been documented in prior studies [18,19]. Yet, unlike these prior studies of physician assessment of adherence performed, the VA providers in this study could electronically review VA pharmacy refill records of medications prescribed at VA at the time of the visit. Despite these available VA pharmacy records, providers still overestimated adherence with antihypertensives. Admittedly, review of such refill records can be time-consuming and hard to interpret as this task requires physicians to examine long lists of medications or to pull up specific graphs of refill patterns for individual medications of interest. Given the multiple chronic conditions and treatment goals that primary care providers must consider in often brief primary care visits, these results suggest that making medication adherence assessments easier and quicker to interpret by providing a simpler objective measure (such as the CMG or a related measure using shorter time intervals known as ReComp [10]) may help increase awareness of self-management challenges by the provider and patient. The availability of such real-time measures could serve as an important step of patient-centered care to improve translation of evidence-based treatment recommendations into improved individual patient outcomes.

Other studies have shown doctors often intensify medications without inquiry and/or awareness of a patient’s poor adherence to prescribed medications [8,20]; yet, ours is the first to demonstrate that providers often intensified BP medications even when providers did suspect serious adherence problems. This implies that providers may have more confidence in their ability to improve BP by intensifying medications rather than addressing adherence issues. Providers may also be simply prioritizing medication intensification rather than evaluating and addressing adherence. For example, providers may be responding to monitored performance measures regarding BP control by intensifying medications because attempting to improve adherence can prove to be difficult and time-consuming. This suggests that better systems approaches are needed to improve medication adherence that do not rely on individual PCPs.

Traditional wisdom teaches that intensifying prescription regimens in non-adherent patients poses additional risk to patients (such as adverse events of dizziness and low BP, should a previously non-adherent patient start taking a higher dose medication regimen) and prior studies have supported lack of better outcomes when medication intensification occurs without concurrent improvement of medication adherences [8,20]. Yet, more recent studies have suggested some improvement in BP control with intensification regardless of the patient’s level of adherence [21]. The potential hazards and benefits of intensifying a regimen in the outpatient setting of prior poor adherence are complicated and difficult to study. For example, it is not uncommon for patients with poor adherence in the outpatient setting to be admitted with elevated blood pressure, then prescribed multiple antihypertensives on a scheduled and observed basis while admitted, leading to significant hypotension with dizziness, increased risk of falling and inadequate renal perfusion - requiring acute treatments to reverse hypotension (i.e., IV fluids, pressors) which are also not without risk. However, in the outpatient setting, sudden perfect adherence of an intensified regimen is likely to occur only in the setting of an additional intervention such as medication administration by family members or visiting nurses. Thus, it is possible that a previously poorly adherent patient would experience a smaller positive impact on blood pressure with an intensification of the regimen (even if only taking 50% of the doses) due to imperfect medication taking at baseline [21]. On the other hand, side effects of outpatient hypotension in response to intensified regimens may not be well reported, particularly if providers do not ask about potential signs and symptoms of hypotension. Of note, the recent study by Rose *et al.*[21] reporting improved BP after intensification in patients with poor adherence did not report patient outcomes such as side effects from hypotension.

Some limitations of the study should be noted. Overall, this study population of VA patients with diabetes and uncontrolled hypertension had high refill adherence, with only 20% of patients with a CMG >20%; this high adherence may generate concerns for generalizability of our results. However, similarly high levels of BP medication adherence have recently been reported for Medicare, managed care, and urban safety net populations [13,21,22]. Also, admittedly, refill adherence is a conservative measure of medication-taking adherence because possession of medications alone does not mean patients are actually taking the medications as prescribed. Likewise, a physician’s assessment of a patient as non-adherent does not refer specifically to the physician’s opinion of the patient’s *refill* adherence, but instead is an assessment of their overall adherence with taking the prescribed medications. Accounting for this, we focused several of our analyses on patients who were first identified as not possessing the medications (CMG ≥ 20%), because it was assumed that if the patient wasn’t refilling the medication then the patient also couldn’t actually be taking the medication as prescribed. This seems a reasonable assumption for VA patients at the time of this study before many of the low-cost generic retail pharmacy programs were developed in late 2006. Another important limitation is that providers were asked to assess patient adherence in a *post*-visit questionnaire; therefore, it is possible that for some patients, the providers had not actually contemplated patient adherence before making medication treatment decisions during the visit such as whether to intensify or not. Yet, this aspect of our study also mirrors clinical practice, as providers are not required to systematically assess adherence prior to implementing management changes.

General limitations using the CMG measure of refill adherence should also be noted. In order for our pharmacy adherence measure to better reflect adherence as indicated by refills the patient requested, we excluded new medications with only one fill from the calculation because first prescriptions in the VA health system are filled automatically without effort required by the patient, and because single fill medications could represent a trial of new medication. Yet, we acknowledge that exclusion of medications with a single fill may decrease detection of the most non-adherent patients who did not fill any antihypertensive medications past the automated first fill. In evaluating for potential impact of missing such patients in our dataset, 558 (15%) of 3695 prescriptions were excluded from our analyses because of no additional refills initiated; yet, most of these patients had additional BP prescriptions that *did* have refills that kept the patient included in the dataset for analysis. Another potential limitation of the CMG measure is loss of information regarding over-supply sources if only outpatient pharmacy data is employed; keeping this in mind, our method for CMG calculation was specifically tailored to account for multiple sources of oversupply by also utilizing inpatient records regarding hospitalizations and discharge medications.

## Conclusions

In summary, despite medication non-adherence being an important and modifiable barrier to improving BP control, PCPs do not accurately detect significant non-adherence by traditional clinical assessment alone. Inaccurate physician assessment of patient adherence has important implications for patient care, including potential harm to patients by continuing to intensify medication regimens despite inconsistent medication taking and missed opportunities for identifying and addressing patient barriers to self-management. Although pharmacy refill adherence is only one aspect of adherence to treatment for hypertension, this type of data is an important objective measure of adherence that may serve as a screening test for detecting and prompting discussion and intervention regarding non-adherence to chronic medications.

## Appendix

Algorithm for Refill Adherence Measure Calculation:Continuous Multiple-Interval Gap (CMG) MeasureOur CMG measure was calculated using the following algorithm, illustrated in Appendix Figure 5:

1. Looking retrospectively 12 months from the patient’s clinic visit, our VA pharmacy record details specific dates of when a medicine was first filled, dates of each fill and refill (noted by thin arrows in Appendix Figure 5), and days of supply the prescription would last if taken as prescribed (shaded boxes in Appendix Figure 5). For the purpose of determining if the patient was already taking a medication chronically prior to the 12 month observation period (and when it would be expected to require refill within the 12 month observation period), prescription records were also evaluated for 90 days prior to the 12 month observation period. For the purpose of determining if medications not refilled recently prior to the time of the appointment were inappropriately late refill requests, or if these medications were not expected to be continued chronically, prescription refill records were also evaluated for 90 days after the clinic visit.

**Figure 5:**
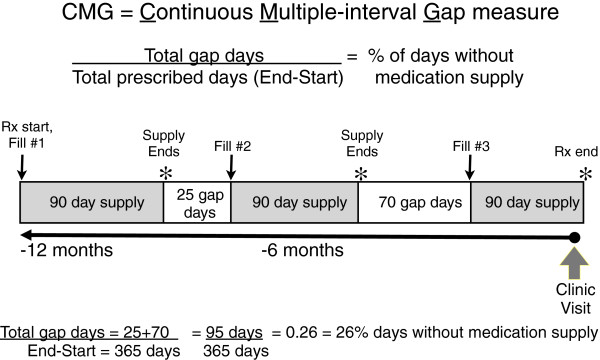
Example of Calculation of Refill Adherence Measure.

2. Next, if the patient was taking the medicine exactly as prescribed at full dose, which date would the patient expected to be without medication (“supply end” noted by * in Appendix Figure 5).

3. Then, all days that occur between the date of supply end and the next refill date are considered to be “gap days” (white boxes in Appendix Figure 5), that is, days where the patient would be expected to be experiencing a gap in medication supply because had not refilled early enough.

4. To generate the CMG for each BP medication class, the total number of gap days for medications in each class was summed, and then divided by the total number of days that patient was prescribed the medications in that class, to yield the following measure:

(none1) $$\begin{array}{l} \frac{\text{Total gap days}}{\text{Total prescribed days}\left(\text{End}-\text{Start}\right)}=\text{CMG}\\ =\text{\% of days without medication supply} \end{array}$$

5. To generate the composite CMG, the total number of gap days for all BP medications (all classes, excluding loop diuretics for reasons explained below) was summed, and then divided by the total number of days the patient was prescribed each medication. Therefore, the composite CMG is not a simple average of the CMG calculated for each BP medication class; instead, it takes into account (and thus is weighted by) the duration of time the patient was prescribed each medication. Thus, a single medication (or a single class) of short duration would not have equal influence as a long-standing chronically prescribed BP medication.

This ratio of total gap days to total prescribed days yields the CMG, which is measure of proportion of days without adequate medication, also known as a ratio of non-possession. For example, a CMG of 0.2 or 20% means the patient refilled the medication in a manner that left 20% of days without an adequate medication supply to take. The CMG can also be interpreted as a proportion of time the patient misses their medications, with a CMG of 20% meaning that on average, the patient missed 20% of the doses, or 1 day or dose in 5. Prior literature has established that a lack of medication possession of  ≥  20% is clinically significant refill non-adherence [14].

In order for our pharmacy adherence measure to better reflect adherence as indicated by refills the patient requested, we excluded new medications with only one fill for the CMG calculation because first prescriptions in the VA health system are filled automatically without effort required by the patient. New medications were identified as having a total expected supply to patient of ≤ 90 days in the study period year, with less than 2 fills. Using the rich and connected VA database of inpatient, outpatient, and pharmacy data, multiple sources of oversupply were also accounted for, including days of hospitalization and early refills which include early medication fills written as part of discharge orders [8,23].

Of note, our refill adherence measure including refill data for all classes of antihypertensives excluding only loop diuretics, because loop diuretics can be taken for reasons such as congestive heart failure or edema with directions and instructions changing frequently by prescribers to treat acute exacerbations (such as increased loop diuretic for 3–4 days, before transition to a stable dose). Thus, the refill record for loop diuretics was not thought to reflect adherence as an antihypertensive treatment. In summary, classes of antihypertensives that were evaluated and included for patients in this study included: angiotensin converting enzyme (ACE) inhibitors, beta blockers, calcium channel blockers, alpha receptor blockers, hydralazine, aldosterone receptor blockers, sympathetic blockers, thiazide diuretics, potassium-sparing diuretics, renin-inhibitors, and minoxidil.

## Competing interests

The authors declare that they have no competing interests.

## Authors’ contributions

JM participated in the study design, performed the data management and statistical analyses, and drafted the manuscript, tables and figures. EK and TH designed the primary study data collection [9] from which the analytic datasets for this manuscript were obtained, provided guidance to JM in the data analysis and interpretation, and design and interpretation of the study presented in this manuscript and provided significant edits to this manuscript. MH provided expertise on interpretation of the refill measure based on prior work [8] and provided significant edits to the manuscript. All authors read and approved the final manuscript.

## Pre-publication history

The pre-publication history for this paper can be accessed here:

http://www.biomedcentral.com/1472-6963/12/270/prepub
